# Electrochemically Stable Cobalt–Zinc Mixed Oxide/Hydroxide Hierarchical Porous Film Electrode for High-Performance Asymmetric Supercapacitor

**DOI:** 10.3390/nano9030345

**Published:** 2019-03-03

**Authors:** Hanbin Yang, Xinqiang Zhu, Enhui Zhu, Gaobo Lou, Yatao Wu, Yingzhuo Lu, Hanyu Wang, Jintao Song, Yingjie Tao, Gu Pei, Qindan Chu, Hao Chen, Zhongqing Ma, Pingan Song, Zhehong Shen

**Affiliations:** School of Engineering, Zhejiang Provincial Collaborative Innovation Center for Bamboo Resources and High-Efficiency Utilization, National Engineering and Technology Research Center of Wood-based Resources Comprehensive Utilization, and Key Laboratory of Wood Science and Technology of Zhejiang Province, Zhejiang A&F University, Hangzhou 311300, China; yanghanbin19970723@163.com (H.Y.); 17816896693@163.com (X.Z.); zeh19970218@163.com (E.Z.); 15957156139@163.com (G.L.); wyt19940822@163.com (Y.W.); tqy033201@163.com (Y.L.); w695670783@163.com (H.W.); 18367416191@163.com (J.S.); taoyingjie123@126.com (Y.T.); pg260251432@163.com (G.P.); 15306587705@163.com (Q.C.); mazq@zafu.edu.cn (Z.M.); pingansong@gmail.com (P.S.)

**Keywords:** hierarchical porous film, cobalt–zinc mixed oxide/hydroxide, supercapacitor, high stability

## Abstract

Construction of electrochemically stable positive materials is still a key challenge to accomplish high rate performance and long cycling life of asymmetric supercapacitors (ASCs). Herein, a novel cobalt–zinc mixed oxide/hydroxide (CoZn-MOH) hierarchical porous film electrode was facilely fabricated based on a cobalt–zinc-based metal–organic framework for excellent utilization in ASC. The as-constructed hierarchical porous film supported on conductive Ni foam possesses a rough surface and abundant macropores and mesopores, which allow fast electron transport, better exposure of electrochemically active sites, and facile electrolyte access and ion diffusion. Owing to these structural merits in collaboration, the CoZn-MOH electrode prepared with a zinc feeding ratio up to 45% at 110 min of heating time (CoZn-MOH-45-110) exhibited a high specific capacitance of 380.4 F·g^−1^, remarkable rate capability (83.6% retention after 20-fold current increase), and outstanding cycling performances (96.5% retention after 10,000 cycles), which exceed the performances of similar active electrodes. Moreover, an ASC based on this CoZn-MOH-45-110 electrode exhibited a high specific capacitance of 158.8 F·g^−1^, an impressive energy density of 45.8 Wh·kg^−1^, superior rate capability (83.1% retention after 50-fold current increase), and satisfactory cycling stability (87.9% capacitance retention after 12,000 cycles).

## 1. Introduction

Over the past decades, great research efforts have been dedicated to explore electrochemical energy storage devices of high performances, where supercapacitor is one of the most attractive types owing to their high power output and preeminent safety [1,2,3]. However, usually, their wider applications are severely hindered by low energy density. Constructing asymmetric supercapacitors (ASCs) to integrate the electrochemical windows of positive and negative electrodes to expand the voltage output range has been proved to be an effective strategy to elevate energy density [4,5,6,7]. Therefore, recent research attentions have paid to develop high-capacitance positive electrode materials to fabricate ASCs [8,9]. Metal oxides and hydroxides are the most widely used positive materials owing to their high theoretic capacitance [10,11,12,13]. Recently, it was found that metal-based mixed oxides/hydroxides (e.g., Co–Ni–Zn ternary oxide [14] and Co–Ni layered double hydroxide [15]) exhibit better capacitance performances compared to simple metal oxides and hydroxides. However, the low rate capability and poor cycling stability still hinder their practical applications in commercial supercapacitors. Therefore, considerable research efforts are being made in attempts to address the issues mentioned above.

In this research, a novel cobalt–zinc mixed oxide/hydroxide (CoZn-MOH) hierarchical porous film electrode was rationally constructed and systematically investigated as a positive electrode for ASC. This hierarchical porous structure was facilely manufactured through an in situ successive hydrolysis, dehydration, and crystallizing process based on the cobalt–zinc-based metal-organic framework (CoZn-MOF). The prefabricated CoZn-MOF arrays supported on nickel foam intentionally served as both metal ion sources and skeleton template. The as-constructed hierarchical porous film consists of abundant macropores and mesopores, which allow facile electrolyte access and ion diffusion within active materials. Meanwhile, the rough surface constructed by interconnected nanoparticles favors better exposure of active sites to participate in electrochemical reactions. Moreover, the hierarchical porous film directly grown on the conductive nickel foam substrate can avoid the electrical resistance of the polymer binder and encourage fast electron transfer to the current collector. Due to these structural merits in collaboration, the CoZn-MOH electrode prepared with a zinc ratio up to 45% at 110 min of heating time (CoZn-MOH-45-110) exhibits a high specific capacitance (*C_s_*) of 380.4 F·g^−1^, remarkable rate capability (83.6% retention after 20-fold current increase), and outstanding cycling performances (96.5% retention after 10,000 cycles), which exceed the performances of electrodes based on similar active materials. When the CoZn-MOH-45-110 electrode served as a positive electrode, the as-fabricated ASC outputs an impressive *C_s_* of 158.8 F·g^−1^ and an impressive energy density of 45.8 Wh·kg^−1^. Moreover, this ASC displays superior rate capability (83.1% retention after 50-fold current increase) and satisfactory cycling stability (87.9% retention after 12,000 cycles), suggesting an optimistic usage potential of the CoZn-MOH-45-110 electrode to produce the next-generation high-performance supercapacitors.

## 2. Materials and Methods

### 2.1. Materials

We purchased 2-methylimidazole (2-MIM, 98% purity), potassium hydroxide (KOH, AR grade), cobalt nitrate hexahydrate (Co(NO_3_)_2_·6H_2_O, 99% purity), zinc nitrate hexahydrate (Zn(NO_3_)_2_·6H_2_O, 99% purity), and anhydrous ethanol (AR grade) from Sinopharm Chemical Reagent Corp. (Shanghai, China). Bamboo fiber powder was donated by Professor Wenbin Yao from Jiyang College of Zhejiang A&F University. All materials were used as received.

### 2.2. Fabrication of CoZn-MOF Supported on Nickel Foam

The CoZn-MOF supported on nickel foam was prepared according to our previous report [12]. In a typical operation process, the commercial nickel foam was pre-cleaned to obtain a clean surface. A 2-MIM (40 mL, 0.4 M) aqueous solution was quickly poured into 40 mL mixed aqueous solution containing Co(NO_3_)_2_·6H_2_O (1.1 mmol) and Zn(NO_3_)_2_·6H_2_O (0.9 mmol), then the clean nickel foam substrates (1 × 4 × 0.1 cm^3^, the upper 1 cm^2^ area was protected by scotch tape) were immersed into this mixture for 4 h to grow the CoZn-MOF under room temperature. After the reaction, the as-obtained sample was washed with deionized H_2_O, and then dried at 60 °C in an oven for 12 h to obtain CoZn-MOF-45 supported on nickel foam. Similarly, other CoZn-MOF-x samples with different feeding molar percentages (x) of zinc ion were also prepared by utilizing a similar process.

### 2.3. Preparation of CoZn-MOH Supported on Nickel Foam

The as-fabricated CoZn-MOF-45 supported on Ni foam was immersed into a mixed solution of 20 mL ethanol and 5 mL H_2_O and kept stationary. The mixed solution was heated to 85 °C to allow an in situ conversion from CoZn-MOF-45 to cobalt–zinc double hydroxide composite (CoZn-MOH-45) on the surface of the Ni substrate. After reaction for 110 min, the resulting product was washed with deionized H_2_O at least three times, and then dried at 60 °C for 12 h to provide a CoZn-MOH-45-110 sample. Similarly, the CoZn-MOH-45-y samples obtained with different reaction times (y) were also prepared by utilizing a similar process. For comparison purposes, the CoZn-MOH-x-110 samples were prepared by treating different CoZn-MOF-x samples via a similar method.

### 2.4. Preparation of AC

The bamboo-fiber-derived activated carbon (AC) was prepared with KOH as the activator through a similar carbonization and activation method to our previous research [16,17]. Typically, the bamboo fiber was first carbonized at 900 °C for 1 h with a heating rate of 3 °C·min^−1^ under N_2_ atmosphere. The as-obtained powder was washed and dried at 100 °C overnight. Then, the powder (2 g) was uniformly mixed with a 45 wt% KOH aqueous solution (8 g), and then heated at 100 °C in a forced convection oven for 8 h to produce a black jelly-like slurry. Then the slurry was heated at 900 °C for 1 h with a heating rate of 3 °C·min^−1^ under N_2_ atmosphere. The resulting material was thoroughly washed and dried at about 100 °C overnight to provide the AC powder.

### 2.5. Fabrication of Supercapacitor Electrodes

The AC electrode was fabricated using a typical process [18,19]: AC powder, polytetrafluorethylene, and conductive acetylene black powder were mixed by milling with a weight ratio of 80:5:15, and then a small amount of deionized H_2_O was added under stirring to create a homogeneous paste. Then, this paste was pressed onto clean nickel foam to fabricate the AC electrode.

### 2.6. Characterization

Scanning electron microscopy (SEM, Zeiss Supra 40, Carl Zeiss Meditec AG, Jena, Germany, accelerating voltage: 5 kV), energy-dispersive X-ray spectroscopy (EDS, JEOL-2100F, JEOL Co., Ltd., Tokyo, Japan), transmission electron microscopy (TEM, JEOL-2100F, JEOL Co., Ltd., Tokyo, Japan, accelerating voltage: 200 kV), X-ray diffractometer (XRD, SmartLab 9, Rigaku Co., Tokyo, Japan, Cu K_α_ radiation, scanning rate: 1°·min^−1^), X-ray photoelectron spectroscopy (XPS, ESCALAB 250Xi, Thermo Fisher Scientific, Waltham, MA, USA), and automated N_2_ gas adsorption–desorption equipment (Micromeritics 3Flex, Micromeritics Instrument Co., Norcross, GA, USA) were used to investigate the properties of samples.

### 2.7. Electrochemical Analysis

The as-prepared CoZn-MOH-x-y samples supported on Ni foam were directly utilized as self-supported electrodes in the electrochemical measurements. The performances of the CoZn-MOH-x-y electrodes and the AC electrode were firstly researched at room temperature in 1 M KOH electrolyte through a typical three-electrode cell system using a saturated calomel electrode (SCE) and platinum (Pt) foil electrodes as the reference and counter electrodes, respectively. The electrochemical properties of an ASC were studied in 1 M KOH electrolyte by employing a two-electrode cell system with the CoZn-MOH-45-110 and the AC electrode as the positive and negative electrodes, respectively. A CHI 760E electrochemical workstation (Shanghai Chenghua Company, Shanghai, China) was employed to record cyclic voltammetry (CV) curves and electrochemical impedance spectra (EIS). The measurement of galvanostatic charge–discharge (CD) curves was conducted on a Land testing system (CT2001A, Wuhan, China).

## 3. Results and Discussion

### 3.1. Morphology and Compositions

**Figure 1:**
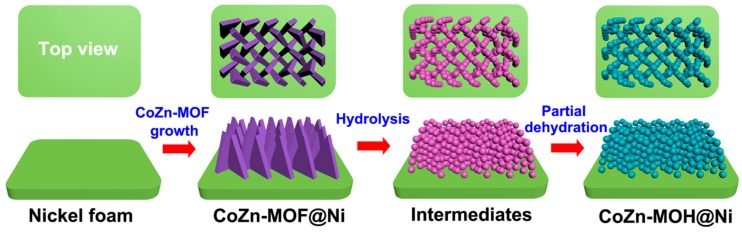
Scheme of the formation process of cobalt–zinc mixed oxide/hydroxide (CoZn-MOH) hierarchical porous film on Ni foam.

The CoZn-MOH hierarchical porous film was prepared by an in situ successive hydrolysis, dehydration, and crystallizing process based on the CoZn-MOF material (Figure 1). First, the CoZn-MOF precursor was pre-grown on an Ni foam substrate via a typical room temperature method as in our previous reports [12]. Then, the as-obtained CoZn-MOF on the nickel foam was placed into a mixed solution of ethanol and H_2_O and kept stationary. The mixed solution was heated to 85 °C to allow water to ionize to produce H^+^. These H^+^ were bound to 2-MIM linkers of CoZn-MOF to form soluble 2-MIMH^+^ in the surrounding solution, resulting in an etching reaction around the CoZn-MOF skeleton to release Co^2+^ and Zn^2+^ [20]. The released Co^2+^ and Zn^2+^ experienced reversible hydrolysis to produce cobalt hydroxide, zinc hydroxide, and H^+^. Because the etching reaction consumed both H^+^ and 2-MIM, the in situ hydrolysis of Co^2+^ and Zn^2+^ was promoted to form more cobalt hydroxide and zinc hydroxide leading to their co-deposition to form cobalt–zinc mixed hydroxide particles. As the process proceeded at 85 °C, the dehydration of pure zinc hydroxide and partial cobalt–zinc-based mixed hydroxides led to the generation of ZnO and zinc cobaltate, respectively. The coexistence of these products resulted in the formation of CoZn-MOH particles. Moreover, these particles gradually crystallized as the reaction time increased.

**Figure 2:**
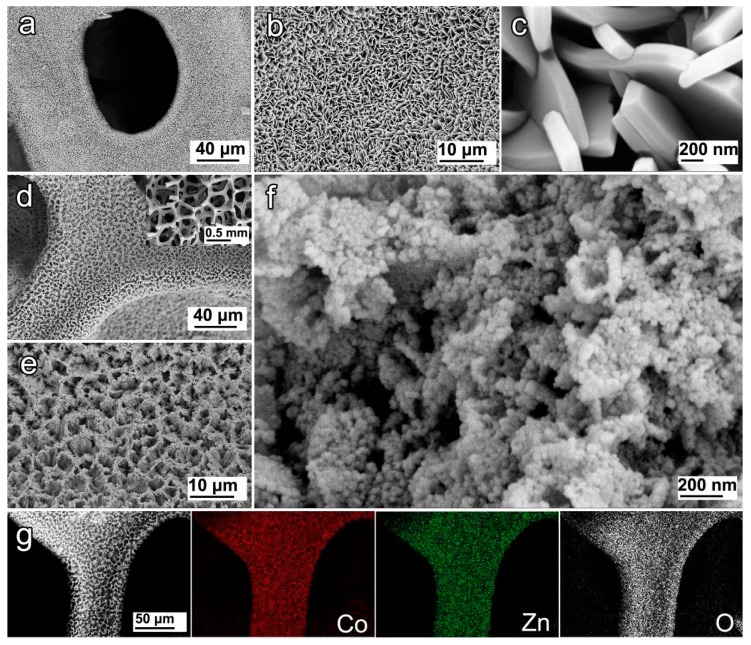
SEM images of (**a–c**) CoZn-MOF-45 (cobalt–zinc-based metal–organic framework) precursor and (**d–f**) as-prepared CoZn-MOH-45-110 supported on nickel foam. The inset of (**d**) is its low-magnification SEM image. (**g**) SEM elemental mapping of CoZn-MOH-45-110 supported on nickel foam.

Figure 2a–c shows the SEM images of the as-obtained CoZn-MOF-45 precursor, which is densely covered on the nickel foam substrate, and possesses a sheet-like array structure. These microsheets had a width of ~1–2.5 μm and a thickness of ~150–250 nm. They interlocked together to form a macroporous structure, thereby favouring the effective penetration of the liquid reagent for the further hydrolysis of CoZn-MOF-45 precursors. After the successive hydrolysis, dehydration, and crystallizing process, a similar macroporous feature was retained for the as-obtained film supported on Ni foam (Figure 2d), and the resulting CoZn-MOH-45-110 material inherited a similar sheet-like skeleton structure (Figure 2e), since the CoZn-MOF-45 sheet-like array served as a sacrificial template and precursor. However, the nanostructure unit of CoZn-MOH-45-110 was found to be a nanoparticle (Figure 2f), unlike its precursor. The size of these nanoparticles was less than 50 nm, and it is estimated that the gap between these nanoparticles would create plentiful mesopores. Moreover, the components of the resulting material were proved to uniformly distribute on nickel foam substrate (Figure 2g).

**Figure 3:**
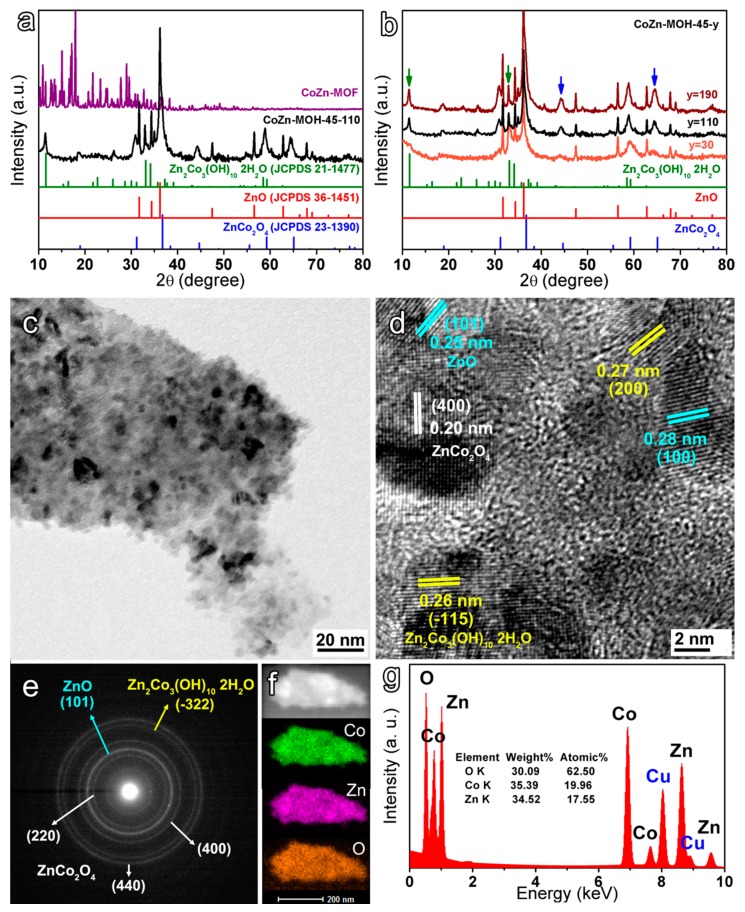
Comparisons of XRD patterns: (**a**) CoZn-MOF-45 and CoZn-MOH-45-110 powders; (**b**) CoZn-MOH-45-y powders; (**c**,**d**) TEM images of CoZn-MOH-45-110; (**e**) the selected area electron diffraction pattern of (c); (**f**) TEM elemental mapping; and (**g**) energy dispersive X-ray spectroscopy (EDS) spectrum of CoZn-MOH-45-110, where the Cu signal comes from the sample holder.

Subsequently, the XRD patterns of the CoZn-MOF-45 precursor and the CoZn-MOH-45-110 sample were researched to investigate the change in the crystalline structure of samples. As displayed in Figure 3a, all diffraction peaks of the CoZn-MOF-45 precursor disappeared in the XRD pattern of the resulting CoZn-MOH-45-110 powder, indicating a complete consumption of the precursor. The as-prepared CoZn-MOH-45-110 exhibited three groups of obvious diffraction peaks, which can be indexed to the plane reflections of Zn_2_Co_3_(OH)_10_•2H_2_O (JCPDS 21-1477), ZnO (JCPDS 36-1451), and ZnCo_2_O_4_ (JCPDS 23-1390) phases, respectively. This indicated that the CoZn-MOH-45-110 sample had a polycrystalline feature and was composed of cobalt–zinc-based hydroxide and oxides: Zn_2_Co_3_(OH)_10_•2H_2_O, ZnO, and ZnCo_2_O_4_. Here, ZnO and ZnCo_2_O_4_ should be the dehydration products of pure zinc hydroxide and cobalt–zinc-based mixed hydroxides, respectively, which formed in the early stage of the reaction. Similarly, when Zn^2+^ was not added for the preparation, the as-obtained sample was identified to be a Co(OH)_2_–Co_3_O_4_ composite (Figure S1a in the Supplementary Materials), wherein the Co_3_O_4_ is a dehydration product of Co(OH)_2_. In order to further analyze the generation and crystallization of three components, the XRD patterns of CoZn-MOH-45-y samples prepared with different reaction times are compared in Figure 3b. Based on this result, one can see that after a short reaction time of 30 min, the resulting product contained moderate crystallinity ZnO and Zn_2_Co_3_(OH)_10_•2H_2_O with a relatively low crystallinity that can be illustrated by the wide (002) and (200) diffraction peaks in Figure S1b and green arrows in Figure 3b. Moreover, the content of ZnCo_2_O_4_ was pretty low. As the reaction time was expanded, the crystallinity of Zn_2_Co_3_(OH)_10_•2H_2_O was improved, and the amount of ZnCo_2_O_4_ was elevated (see the (400) and (440) diffraction peaks in Figure S1b and blue arrows in Figure 3b). When the reaction time increased to 190 min, the content and crystallinity of ZnCo_2_O_4_ reached the highest values, and the Zn_2_Co_3_(OH)_10_•2H_2_O and ZnO also possessed relatively high crystallinity. TEM images were further studied to acquire the detailed microstructure of the as-prepared CoZn-MOH-45-110 product (Figure 3c–e). It was found that its sheet-like skeleton structure consisted of small nanoparticles with a size of about ~5–20 nm (Figure 3c), which is consistent with the SEM results. Moreover, in a representative high-resolution TEM image (Figure 3d), the observed and calculated d-values of 0.26 and 0.27 nm were indexed to the d-spacing of (–115) and (200) facets of Zn_2_Co_3_(OH)_10_•2H_2_O, respectively. The clear fringe spacings of 0.25 and 0.28 nm were assigned to the (101) and (100) planes of the ZnO component, respectively. Moreover, the lattice fringe with an interplanar spacing of 0.20 nm was associated with the (400) plane of ZnCo_2_O_4_ phase. The selected-area electron diffraction (SAED) pattern in Figure 3e further confirmed the polycrystalline nature of the as-prepared CoZn-MOH-45-110 sample, and the diffraction rings were indexed to the (101) plane of ZnO; (–322) plane of Zn_2_Co_3_(OH)_10_•2H_2_O; and (220), (400), and (440) planes of ZnCo_2_O_4_. These findings were consistent with the previous XRD results. After that, the elemental composition of a single sheet-like skeleton structure was investigated via EDS. The single sheet-like skeleton was proved to possess a uniform elemental composition (Figure 3f). The atomic percentages of Co and Zn were calculated to be 19.96% and 17.55% (Figure 3g), giving rise to a Co/Zn ratio of 1.14:1, which was close to the original Co/Zn feeding molar ratio (1:22) for the precursor preparation.

**Figure 4:**
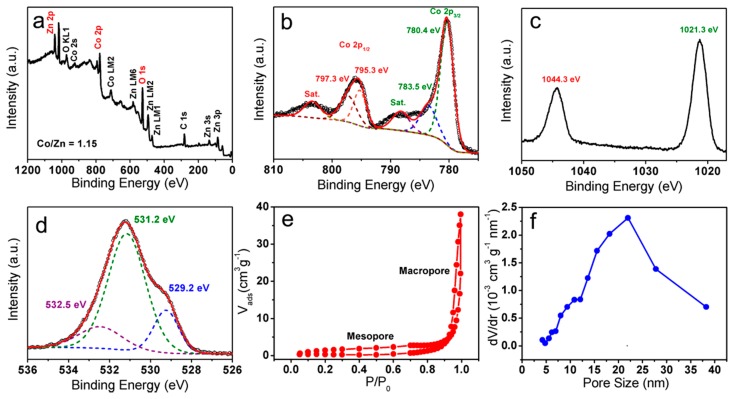
(**a**) Full X-ray photoelectron spectroscopy (XPS) scan, (**b**) Co 2p, (**c**) Zn 2p, and (**d**) O 1s XPS scans of the CoZn-MOH-45-110 sample. (**e**) N_2_ (77 K) adsorption/desorption isotherms and (**f**) Barrett-Joyner-Halenda (BJH) pore size distribution curves of CoZn-MOH-45-110 sample supported on nickel foam.

To study the surface chemical state of as-obtained CoZn-MOH-45-110 product, its XPS was investigated. From Figure 4a, the signals of Co, Zn, and O elements are seen as expected. Moreover, we found that the atomic ratio of Co to Zn can be calculated as 1.15:1 based on the Co 2p and Zn 2p peaks. This ratio is consistent with the value (1.14:1) obtained from the previous EDS result, indicating an excellent composition uniformity of the interior and the surface of our CoZn-MOH-45-110 material. The high-resolution Co 2p, Zn 2p, and O 1s XPS scans of CoZn-MOH-45-110 were further researched to reveal the valence state of cobalt and zinc ions as well as the types of oxygen-containing species. The deconvolutions of the Co 2p XPS spectrum (Figure 4b) displayed a pair of fitting peaks located at 797.3 and 783.5 eV, respectively, which were associated with Co^2+^ [21]. Another pair of fitting peaks centered at 795.3 and 780.4 eV indicated the existence of Co^3+^ [22]. The Zn 2p spectrum in Figure 4c shows two strong peaks at 1044.3 and 1021.3 eV, which were indexed to the Zn 2p_3/2_ and Zn 2p_1/2_ spin-orbit peaks of Zn^2+^, respectively [23]. Furthermore, the O 1s XPS spectrum in Figure 4d revealed the existence of adsorbed O species (532.5 eV), metal hydroxides (531.2 eV), and metal oxide (529.2 eV) [24]. These XPS analysis results further confirmed the generation of Zn_2_Co_3_(OH)_10_•2H_2_O, ZnO, and ZnCo_2_O_4_ in the final product. Considering that the porous feature was found by previous SEM observation, the N_2_ adsorption/desorption measurement was employed to investigate the detailed pore structure of the as-obtained CoZn-MOH-45-110 sample. Figure 4e displays the coexistence of mesopores and macropores in the CoZn-MOH-45-110 sample supported on the nickel foam. The average pore size was found to be 22 nm (Figure 4f). The specific surface area and total pore volume of pure CoZn-MOH-45-110 powder were further calculated to be 112 m²·g^−1^ and 0.311 cm^3^·g^−1^, respectively (Figure S2). This pore-rich feature of our CoZn-MOH-45-110 sample would promote electrolyte access and ion diffusion. Thus, it could provide excellent electrochemical performances, when employed as the supercapacitor electrode active material. 

### 3.2. Electrochemical Properties of Electrodes

**Figure 5:**
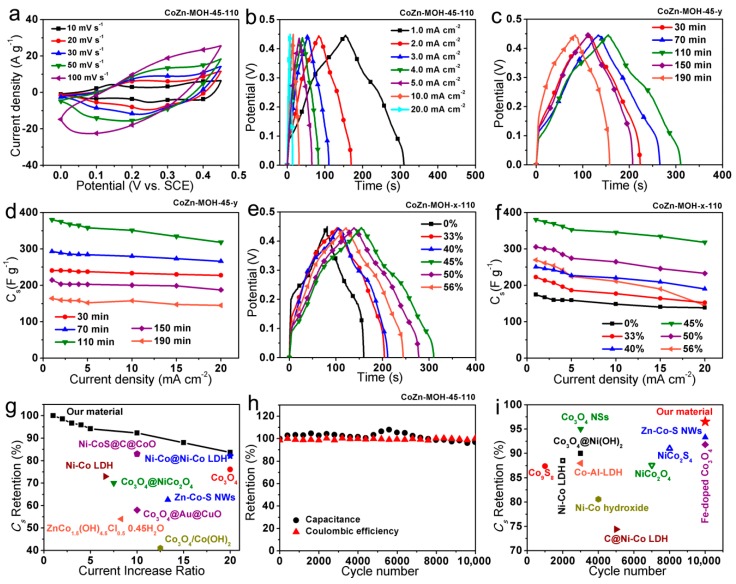
(**a**) Cyclic voltammetry (CV) curves, (**b**) charge–discharge (CD) curves, and (**h**) cycling performances of CoZn-MOH-45-110 electrode. Comparisons of (**c**) CD curves at 1 mA·cm^−2^ and (**d**) specific capacitance (*C_s_*) of CoZn-MOH-45-y electrodes prepared with different times. Comparisons of (**e**) CD curves at 1 mA·cm^−2^ and (**f**) *C_s_* of CoZn-MOH-x-110 electrodes prepared with different feeding molar percentages of zinc ion. Comparisons of (**g**) rate capabilities and (**i**) cycling performances of our CoZn-MOH-45-110 electrode and similar electrodes.

The electrochemical performances of the as-fabricated CoZn-MOH electrodes were first investigated in a three-electrode cell system. Figure 5a describes the representative CV curves of CoZn-MOH-45-110 electrodes at various scan rates, in which two pairs of redox peaks were related to the faradaic redox reactions of Co^2+^/Co^3+^ and Co^3+^/Co^4+^ conversions [25,26]. In Figure 5b, the CD curves of this electrode display slight bending, indicating a typical pseudocapacitive feature. In order to explore the effect of reaction time on the performances of as-prepared electrodes, the CD curves at 1 mA·cm^−2^ of CoZn-MOH-45-y electrodes prepared with different times are compared in Figure 5c. One can see that the CD curves of these electrodes have similar pseudocapacitive characteristic but different CD times. The CD time increased first and then declined with the increment of reaction time, where the longest CD time was obtained for the electrode prepared with 110 min. Based on the corresponding CD curves, the *C_s_* of five CoZn-MOH-45-y electrodes were calculated and listed in Figure 5d. Their *C_s_* values exhibited similar variation at different current densities. The CoZn-MOH-45-110 electrode achieved the highest *C_s_* of nearly 400 F·g^−1^. In order to discover the reasons for this performance variation, the EDS spectra, XRD patterns, and SEM images of these CoZn-MOH-45-y samples were compared and analyzed. The EDS spectra in Figure S3a show that no nitrogen signal can be detected in the CoZn-MOH-45-30 sample, indicating the conversion of CoZn-MOF-45 precursor was completed within 30 min, which is consistent with the XRD result in Figure 3b. As discussed previously, the crystallinity of the three components and the content of ZnCo_2_O_4_ in the CoZn-MOH-45-y was elevated by prolonging the treatment time (Figure 3b and Figure S1b), thus the conductivity of the material was improved. As a result, the equivalent series resistance (R_s_) of the electrode based on the CoZn-MOH-45-y material was decreased when the preparation time was extended from 30 to 110 min (Figure S3b). The preceding performance improvement with increasing preparation time should benefit from this R_s_ decline. However, further prolonging the synthesis time resulted in an excessive increment in the size and amount of nanoparticle units, as shown in Figure S4. This may weaken the electron transport capability between the active material nanoparticles, thereby there was an increase in R_s_ with the expansion of reaction time from 110 to 190 min (Figure S3b). Moreover, further prolonging the heating time also causes an excessive elevation of the crystallinity of active materials, which may depress the diffusion of electrolyte ions into the active materials. Therefore, the electrodes exhibited decreased capacitance performances. The CoZn-MOH-45-110 material synthesized with a moderate heating time of 110 min had an appropriate crystallinity (Figure 3b) and the best electron transport ability (Figure S3b), and thereby delivered the best capacitance performances (Figure 5c,d).

In addition to the reaction time, the influence of the feeding molar percentage of zinc ion during the precursor preparation on the performances of as-fabricated CoZn-MOH electrodes was also investigated. Figure 5e compares the CD curves of CoZn-MOH-x-110 electrodes prepared with different feeding molar percentages of zinc ion. It was observed that all CoZn-MOH-x-110 electrodes had longer CD time than the electrode based on pure cobalt based mixed oxide/hydroxide (Co-MOH-0-110), indicating better capacitance performances of as-fabricated CoZn-MOH-x-110 electrodes. Moreover, the CD time of CoZn-MOH-x-110 electrode increased first and then declined as the Zn feeding ratio increased, and reached the maximum value at 45% of Zn feeding ratio. Derived from the related CD curves, the *C_s_* values of electrodes were obtained and compared in Figure 5f. The *C_s_* of CoZn-MOH-45-110 electrodes were found to be much higher than that of Co-MOH-0-110 electrode, which should be attributed to the difference of their electrochemical resistance. In Figure S5a, no obvious interfacial charge transfer resistance (R_ct_) can be observed for the CoZn-MOH-45-110 electrode, but the Co-MOH-0-110 electrode had a large R_ct_, which was not conducive to charge transfer and thus reduced the capacitance output. Figure 5f also illustrates that the *C_s_* values of CoZn-MOH-x-110 electrode increased at first and then decreased with increasing Zn feeding ratio, which is similar to the comparison result of CD curves. The comparison of Nyquist plots in Figure S5b indicates the increasing of Zn content can reduce the R_s_ of as-prepared electrodes, thus their *C_s_* can be improved. However, very high Zn contents (e.g., 50% and 60%) caused the collapse of the microstructure (Figure S6) and reduced the electrochemical active sites (Co atoms), hence the capacitance output was reduced. The CoZn-MOH-45-110 electrode obtained with 45% of Zn feeding ratio had appropriate electron transport ability and ideal microstructure, and therefore delivered the highest *C_s_* values of 380.4, 374.9, 367.7, 364.7, 358.2, 351.1, 334.7, and 318.2 F·g^−1^ at different CD current densities of 1, 2, 3, 4, 5, 10, 15, and 20 mA·cm^−2^, respectively. By calculation based on these values, the *C_s_* can retain 83.6% of its original value after a 20-times growth of the CD current density, manifesting an outstanding rate capability of CoZn-MOH-45-110 electrode. Moreover, the rate performances of this CoZn-MOH-45-110 electrode were superior to those of similar electrodes (Figure 5g), such as Co_3_O_4_ [27], Zn–Co–S nanowires (NWs) [9], Co_3_O_4_@NiCo_2_O_4_ [28], Co_3_O_4_@Au@CuO hybrid NWs [29], ZnCo_1.5_(OH)_4.5_Cl_0.5_·0.45H_2_O [30], Ni–Co layered double hydroxide (LDH) [31], Co_3_O_4_/Co(OH)_2_ [32], Ni–Co@Ni–Co LDH [21], and Ni–CoS@C@CoO [33]. Such superior rate performance of the CoZn-MOH-45-110 electrode should be associated with its unique structural features, namely: (i) The as-constructed hierarchical porous film consisting of abundant macropores and mesopores, which allow facile electrolyte access and ion diffusion within active materials. (ii) The rough surface constructed by interconnected nanoparticles, which favors better exposure of active sites to participate in electrochemical reactions. (iii) The hierarchical porous film directly grown on the conductive Ni foam substrate, which avoids the electrical resistance of the polymer binder and encourages quick electron transport to the current collector. These merits strongly support the capacitance delivery under high CD current densities, thereby realizing the outstanding rate capabilities.

In addition to excellent rate performances, this CoZn-MOH-45-110 electrode also exhibited satisfactory cycling performances (Figure 5h). Nearly 96.5% of initial *C_s_* was maintained after 10,000 continuous CD cycles at the current density of 3 mA·cm^−2^. Moreover, the Coulombic efficiency value was above 99% during the whole CD cycling measurement, indicating outstanding electrochemical reversibility of this electrode. As depicted in Figure 5i, this cycling stability also significantly outperformed the levels of some related supercapacitor electrodes, including Co_3_O_4_@Ni(OH)_2_ [34], Zn–Co–S NWs [9], Co_3_O_4_ nanosheets (NSs) [35], Fe-doped Co_3_O_4_ [36], Co–Al–LDH [37], C@Ni–Co LDH [38], Ni–Co hydroxide [39], Ni–Co LDH [40], Co_9_S_8_ [41], NiCo_2_S_4_ [42], and NiCo_2_O_4_ [43]. The nanoparticle feature of the microstructure units and the appropriate crystallinity of CoZn-MOH-45-110 material were likely significant contributory factors to this superb cycling stability. This structural characteristic could avoid the deformation and flaking off of active materials during the repeated charge and discharge process. Based upon the above performances, this CoZn-MOH-45-110 hierarchical porous film electrode was anticipated as the qualified positive electrode to fabricate high-performance ASCs.

### 3.3. Electrochemical Performances of CoZn-MOH-45-110//AC ASC

**Figure 6:**
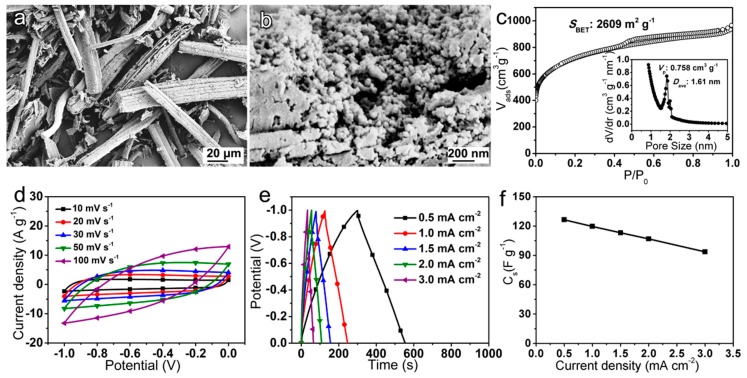
(**a**,**b**) SEM images, (**c**) N_2_ (77 K) adsorption/desorption isotherms and BJH pore size distribution curves (the inset), (**d**) CV curves, (**e**) CD curves, and (**f**) *C_s_* of the as-prepared activated carbon (AC) material.

In order to achieve the construction of an ASC based on the best CoZn-MOH-45-110 electrode, a bamboo-fiber-derived activated carbon (AC) was prepared for usage as the negative material. This AC material retained the fiber-like shape of bamboo fiber (Figure 6a). The surface of the AC fiber showed visible pore structure due to the activation of KOH (Figure 6b). The specific surface area, total pore volume, and average pore size were calculated to be 2609 m^2^·g^−1^, 0.758 cm^3^·g^−1^, and 1.61 nm, respectively (Figure 6c). An electrode based on this AC material as the active material exhibited typical electric double-layer capacitance behavior in its CV and CD curves (Figure 6d,e). By calculation from CD curves (Figure 6e), the *C_s_* of AC-electrode was found to be 126.5 F·g^−1^ at 0.5 mA·cm^−2^ (Figure 6f). In view of the excellent capacitance performances of the CoZn-MOH-45-110 electrode (within 0.0−0.45 V) and the AC electrode (within −1.0−0.0 V), a CoZn-MOH-45-110//AC ASC was successfully assembled by balancing the mass of CoZn-MOH-45-110 and AC based on their *C_s_* values calculated from the CV curves at 10 mV·s^−1^. 

**Figure 7:**
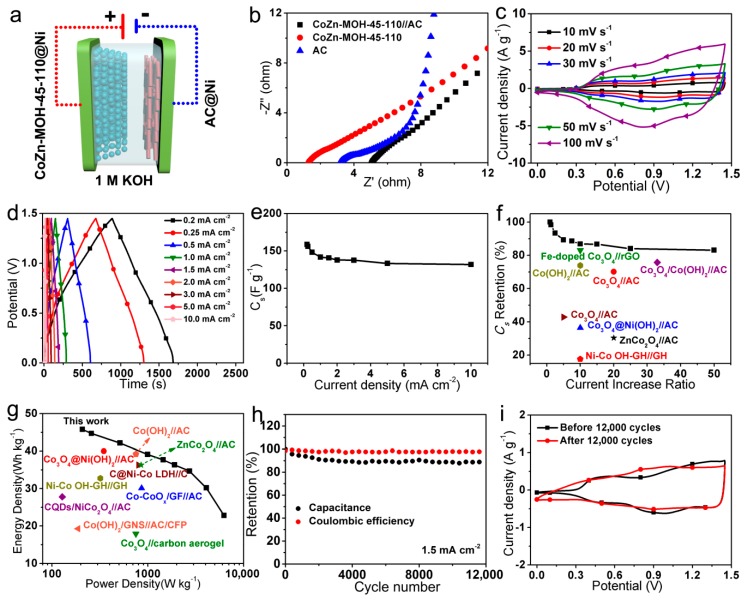
(**a**) Schematic diagram, (**c**) CV curves, (**d**) galvanostatic CD curves, (**e**) *C_s_*, (**h**) cycling performances, and (**i**) CV curves at 10 mV·s^−1^ before and after the cycling performance test of as-assembled CoZn-MOH-45-110//AC asymmetric supercapacitor (ASC). (**b**) Nyquist plots of individual CoZn-MOH-45-110 electrode, AC electrode, and ASC. Comparisons of (**f**) rate capabilities and (**g**) energy densities of this ASC and similar ASCs.

The electrochemical properties of this ASC were explored via a two-electrode cell configuration as shown in Figure 7a. As expected, the R_s_ value of this ASC was approximately equal to the sum of the values of the CoZn-MOH-45-110 and the AC electrodes (Figure 7b). Moreover, compared to the curve of the CoZn-MOH-45-110 electrode, the Nyquist plot of the ASC was more vertical at relatively low frequencies, indicating its enhanced capacitive feature owing to the capacitance contribution of the AC negative electrode. Figure 7c depicts the CV curves of this ASC at different sweep rates, which exhibit the double contribution of pseudocapacitance (CoZn-MOH-45-110 electrode) and electric double-layer capacitance (AC electrode). Derived from the CD curves in Figure 7d or Figure S7, the as-fabricated ASC was found to have a stable discharge *C_s_* of 158.8, 156.1, 148.3, 141.8, 140.7, 137.9, 137.7, 133.4, and 131.9 F·g^−1^ at current densities of 0.2, 0.25, 0.5, 1, 1.5, 2, 3, 5, and 10 mA·cm^−2^, respectively (Figure 7e). Based on these *C_s_* values, nearly 84.0% and 83.1% of the original *C_s_* was retained when the current density increased 25 and 50 times, respectively, demonstrating the superb rate capabilities. More importantly, these rate capabilities were much better than that of many similar ASCs (Figure 7f), including Co_3_O_4_//AC [27], Co_3_O_4_@Ni(OH)_2_//AC [34], Fe-doped Co_3_O_4_//3D reduced graphene oxide (rGO) [36], Co_3_O_4_/Co(OH)_2_//AC [32], Co_3_O_4_//AC [44], Co(OH)_2_//AC [45], ZnCo_2_O_4_//AC [23], and Ni–Co OH–graphene hydrogel (GH)//GH [46]. It is believed that the outstanding high-rate output capability of the CoZn-MOH-45-110 electrode provides powerful support to the excellent rate performance of this ASC. From the obtained *C_s_* values, the energy densities of ASC were calculated to be 45.8, 44.7, 42.2, 39.1, 37.7, 36.4, 34.7, 30.2, and 22.8 Wh·kg^−1^ recorded at average power densities of 208, 258, 512, 994, 1447, 1899, 2722, 4077, and 6227 W·kg^−1^, respectively. Among these data, the maximum energy density (45.8 Wh·kg^−1^ at 208 W·kg^−1^) clearly surpassed that of similar ASCs reported (Figure 7g), such as Co_3_O_4_@Ni(OH)_2_//AC [34], Co–CoO_x_/graphite felt (GF)//AC [47], Co_3_O_4_//carbon aerogel [48], carbon quantum dots (CQDs)/NiCo_2_O_4_//AC [49], Co(OH)_2_/ graphene nanosheet (GNS)//AC/carbon fiber paper (CFP) [50], C@Ni–Co LDH//C [38], Ni–Co OH–GH//GH [46], ZnCo_2_O_4_//AC [23], and Co(OH)_2_//AC [45]. In addition, a long-term repeated CD test at 1.5 mA·cm^−2^ was applied to evaluate the cycling stabilities of the as-assembled CoZn-MOH-45-110//AC ASC. Figure 7h reveals that the *C_s_* underwent a slow reduction during the whole cycling period, and completed the preservation of 87.9% initial capacitance after 12,000 cycles, indicating a satisfactory cycling life. In addition, this ASC had a stable Coulombic efficiency value of 98% during the cycling test process, suggesting nice electrochemical energy conversion efficiency. 

After this performance measurement, the microstructure and CV signal of the CoZn-MOH-45-110 electrode were also researched to understand the reasons for the capacitance decline. As displayed in Figure S8, there was a slight increase in the size of nanoparticle units, and some macropores of the electrode surface were closed, which may have reduced the electrochemical activity and thereby weakened the capacitance delivery. In addition, no obvious redox peak was seen in the CV curve of the CoZn-MOH-45-110 electrode after the cycling test (Figure 7i), further confirming the attenuation of electrochemical activity. Nevertheless, the resulting cycling stability (87.9% retention after 12,000 cycles) was still comparable to the performances of some typical ASCs, such as Ni–Co OH–GH//GH (85% retention after 5000 cycles) [46], Co_3_O_4_//carbon aerogel (85% retention after 1000 cycles) [48], Ni–Co LDH//rGO (82% retention after 5000 cycles) [31], Ni(OH)_2_–MnO_2_//rGO (76% retention after 3000 cycles) [51], and Ni(OH)_2_–MnO_2_–rGO//freeze-dried rGO (75% retention after 2000 cycles) [52].

## 4. Conclusions

In this research, a novel CoZn-MOH hierarchical porous film electrode was facilely made through an in situ successive hydrolysis, dehydration, and crystallizing process based on CoZn-MOF. The as-constructed hierarchical porous film consisted of abundant macropores and mesopores, which allowed facile electrolyte access and ion diffusion within active materials. Meanwhile, the rough surface constructed by interconnected nanoparticles favored better exposure of active sites to participate in electrochemical reactions. Moreover, the hierarchical porous film directly grown on the conductive Ni foam substrate avoided the electrical resistance of the polymer binder and encouraged quick electron transport to the current collector. In addition, the appropriate oxide/hydroxide ratio and the zinc addition reduced the R_s_ and R_ct_ of electrode, respectively. Owing to these structural and compositional merits in collaboration, the as-prepared CoZn-MOH-45-110 electrode exhibited a high *C_s_* of 380.4 F·g^−1^, remarkable rate capability (83.6% retention after 20-fold current increase), and outstanding cycling performances (96.5% retention after 10,000 cycles), which exceeded the performances of electrodes based on Co-MOH-0-110 material and other similar active materials. When this CoZn-MOH-45-110 electrode served as the positive electrode, and a bamboo-fiber-derived AC electrode acted as the negative electrode, the as-fabricated ASC delivered a *C_s_* of 158.8 F·g^−1^ and an impressive energy density of 45.8 Wh·kg^−1^. Moreover, this ASC also exhibited superior rate capability (83.1% retention after 50-fold current increase) and satisfactory cycling stability (87.9% retention after 12,000 cycles). These performances shine among the similar ASCs, therefore, our CoZn-MOH-45-110 electrode enjoys an optimistic usage potential to produce the next-generation high-performance supercapacitors.

## Supplementary Materials

The following are available online at https://www.mdpi.com/2079-4991/9/3/345/s1, Figure S1: (**a**) XRD pattern of pure cobalt-based mixed oxide/hydroxide powder. (**b**) Comparisons of XRD patterns of CoZn-MOH-45-y powders within selected diffraction angle ranges; Figure S2: (**a**) N_2_ (77 K) adsorption/desorption isotherms and (**b**) BJH pore size distribution curves of CoZn-MOH-45-110 powder; Figure S3: Comparisons of (**a**) EDS spectra and (**b**) Nyquist plots of CoZn-MOH-45-y samples supported on nickel foam prepared with different reaction times; Figure S4: SEM images of CoZn-MOH-45-y supported on nickel foam prepared with different reaction times: (**a**,**b**) 30, (**c**,**d**) 70, (**e**,**f**) 150, and (**g**,**h**) 190 min; Figure S5: Comparisons of Nyquist plots: (**a**) Co-MOH-0-110 and CoZn-MOH-45-110 electrodes, (**b**) CoZn-MOH-x-110 electrodes prepared with different feeding molar percentage of zinc ion; Figure S6: SEM images of CoZn-MOH-x-110 supported on nickel foam prepared with different feeding molar percentages of zinc ion: (**a**) 0%, (**b**) 33%, (**c**) 40%, (**d**) 45%, (**e**) 50%, and (**f**) 56%; Figure S7: Galvanostatic CD curves of as-assembled CoZn-MOH-45-110//AC ASC; Figure S8: (**a**,**b**) SEM images of CoZn-MOH-45-110 electrode after the cycling performance test.
