# Supplementary material for: Electrochemically Stable Cobalt–Zinc Mixed Oxide/Hydroxide Hierarchical Porous Film Electrode for High-Performance Asymmetric Supercapacitor
Source: Nanomaterials (Basel). 2019 Mar 3;9(3):345. doi: 10.3390/nano9030345 (PMC6474017; doi:10.3390/nano9030345)
Supplement: Supplementary file 1 [file nanomaterials-09-00345-s001.pdf]

Supporting Information

Electrochemically Stable Cobalt-Zinc Mixed Oxide/Hydroxide Hierarchical Porous Film Electrode for High-performance Asymmetric Supercapacitor

Hanbin Yang, Xinqiang Zhu, Enhui Zhu, Gaobo Lou, Yatao Wu, Yingzhuo Lu, Hanyu Wang, Jintao Song, Yingjie Tao, Gu Pei, Qindan Chu, Hao Chen *, Zhongqing Ma, Pingan Song and Zhehong Shen *

School of Engineering, Zhejiang Provincial Collaborative Innovation Center for Bamboo Resources and High-Efficiency Utilization, National Engineering and Technology Research Center of Wood-based Resources Comprehensive Utilization, and Key Laboratory of Wood Science and Technology of Zhejiang Province, Zhejiang A&F University, Hangzhou 311300, China; yanghanbin19970723@163.com (H.Y.); 17816896693@163.com (X.Z.); zeh19970218@163.com (E.Z.); 15957156139@163.com (G.L.); wyt19940822@163.com (Y.W.); tqy033201@163.com (Y.L.); w695670783@163.com (H.W.); 18367416191@163.com (J.S.); taoyingjie123@126.com (Y.T.); pg260251432@163.com (G.P.); 15306587705@163.com (Q.C.); mazq@zafu.edu.cn (Z.M.); pingansong@gmail.com (P.S.)

* Correspondence: haochen@zafu.edu.cn (H.C.); zhehongshen@zafu.edu.cn (Z.S.); Tel.: + 86-0571-63741609 (H.C. & Z.S.)

The calculation of d-spacing based on XRD patterns:

The layer distance (d) is determined from the Bragg equation: $n\lambda = 2d\sin\theta$, where n equals an integer, λ is the wavelength of X-ray beams, 2θ is the scattering angle corresponding to the given diffraction peak. Here, n = 1, λ = 0.15406 nm. Therefore, d = 0.15406 nm/2sinθ.

For example, the 2θ values of (-115) and (200) facets of $Zn_2Co_3(OH)_{10}\bullet 2H_2O$ are found to be 34.195 and 33.152 degree, respectively, based on Figure 3a and the standard XRD pattern of $Zn_2Co_3(OH)_{10}\bullet 2H_2O$ (JCPDS 21-1477). Thus, the θ values of (-115) and (200) facets are 17.098 and 16.576 degree, respectively. Based on d = 0.15406 nm/2sinθ, the d_{-115} and d_{200} are calculated to be 0.26 and 0.27 nm, respectively.

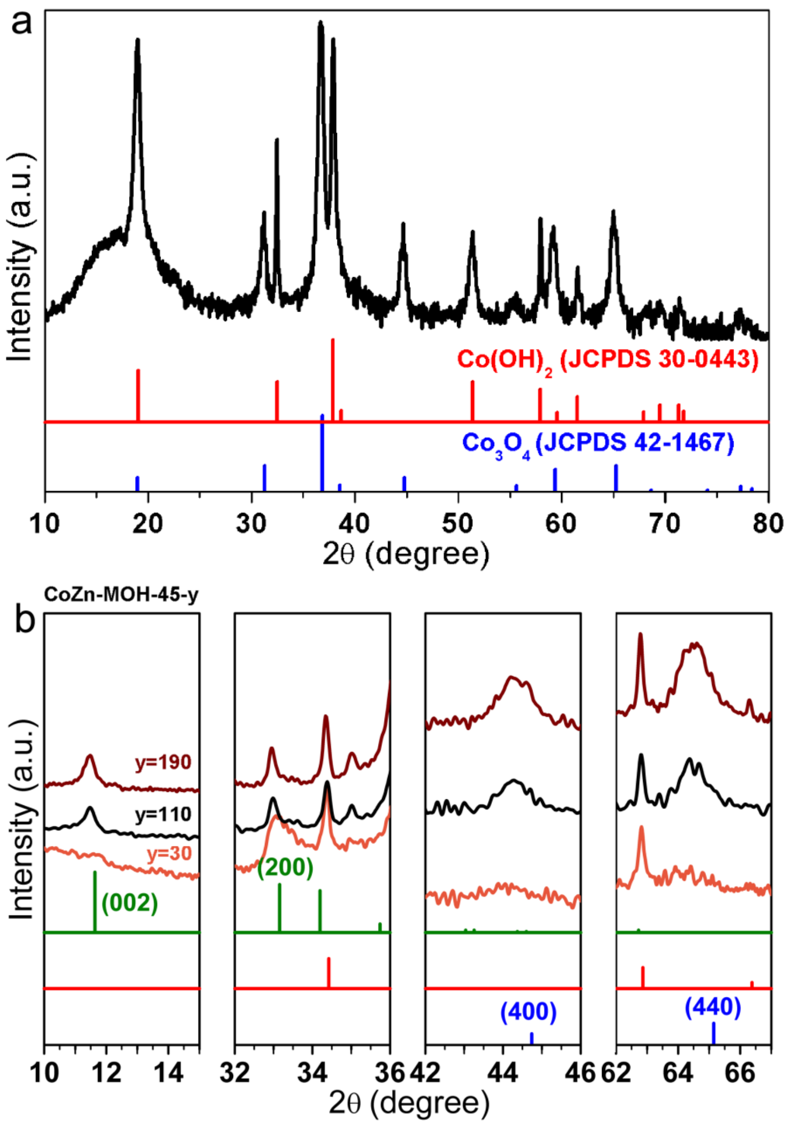

Figure S1. (**a**) XRD pattern of pure cobalt based mixed oxide/hydroxide powder. (**b**) Comparisons of XRD patterns of CoZn-MOH-45-y powders within selected diffraction angle ranges.

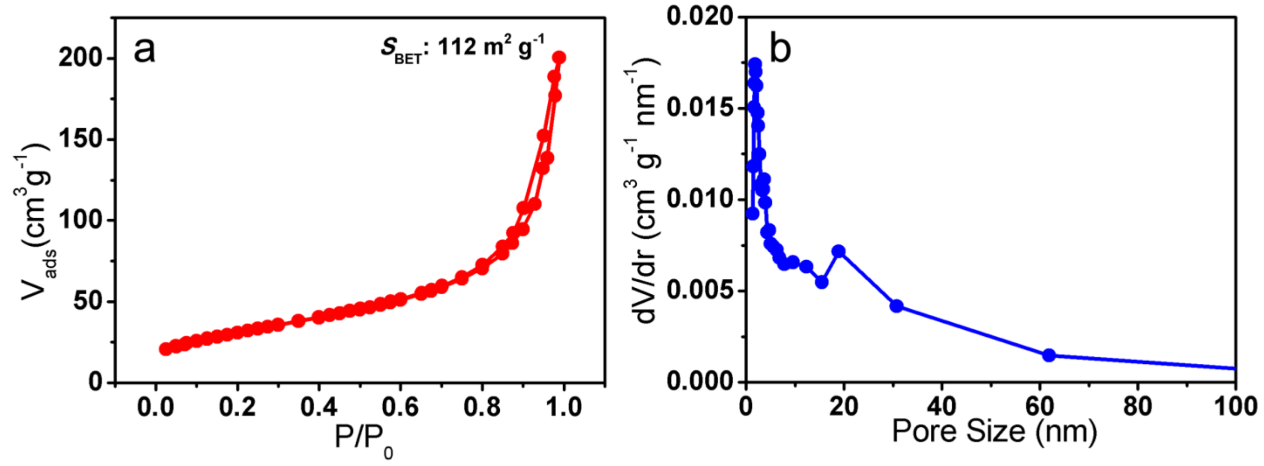

Figure S2. (**a**) N_2 (77 K) adsorption/desorption isotherms and (**b**) BJH pore size distribution curves of CoZn-MOH-45-110 powder.

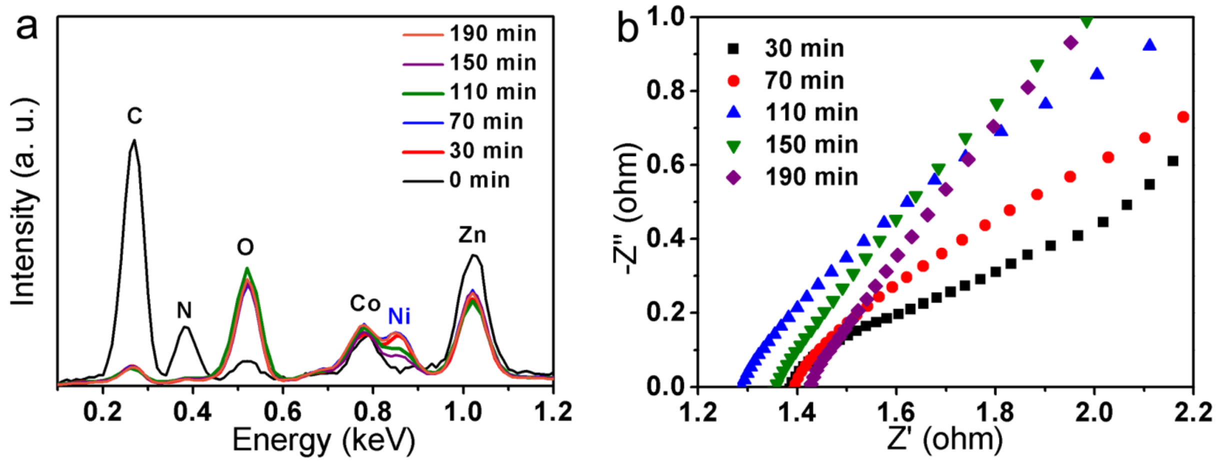

Figure S3. Comparisons of (**a**) EDS spectra and (**b**) Nyquist plots of CoZn-MOH-45-y samples supported on nickel foam prepared with different reaction time.

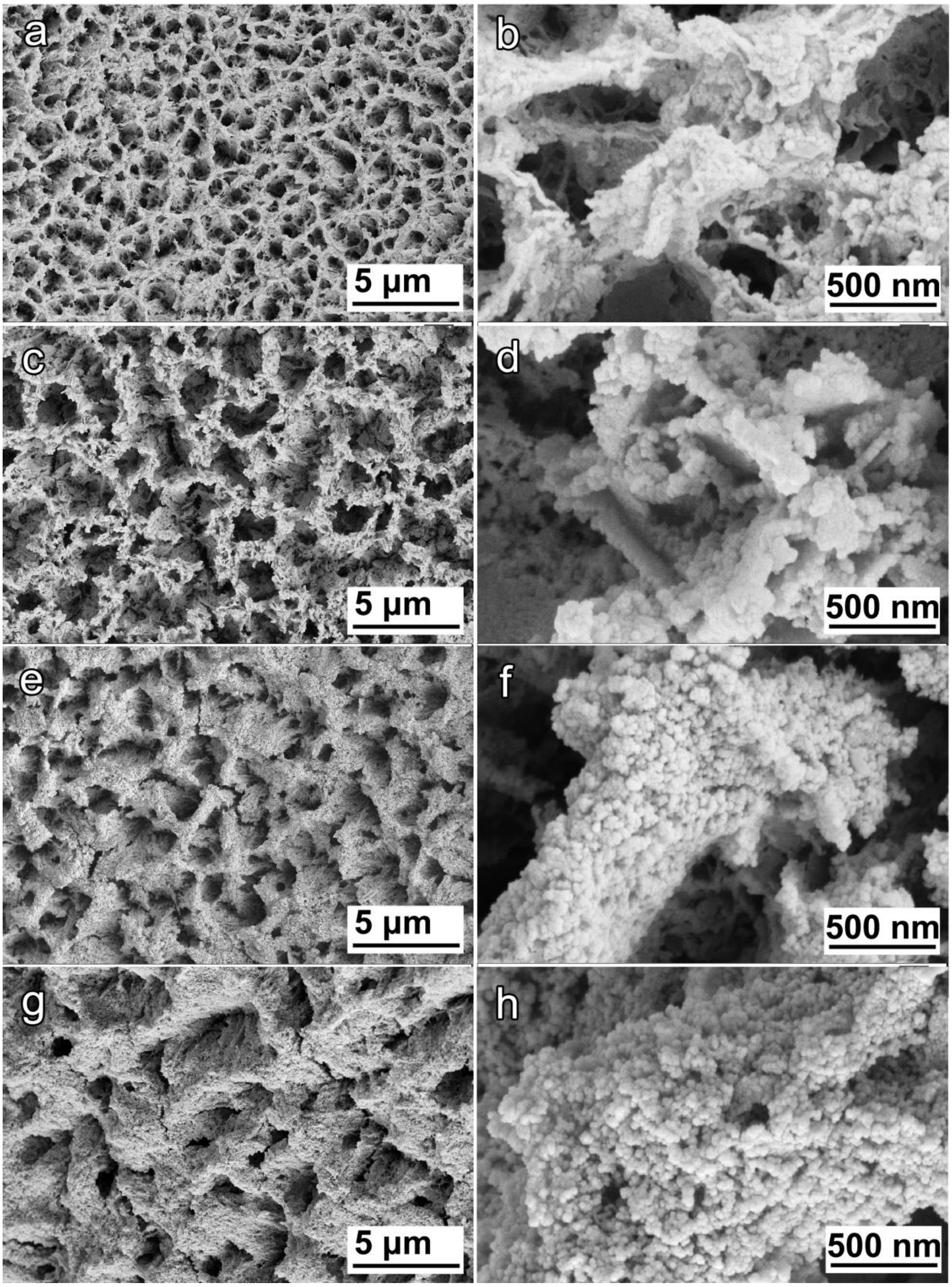

Figure S4. SEM images of CoZn-MOH-45-y supported on nickel foam prepared with different reaction time: (**a**,**b**) 30, (**c**,**d**) 70, (**e**,**f**) 150, and (**g**,**h**) 190 min.

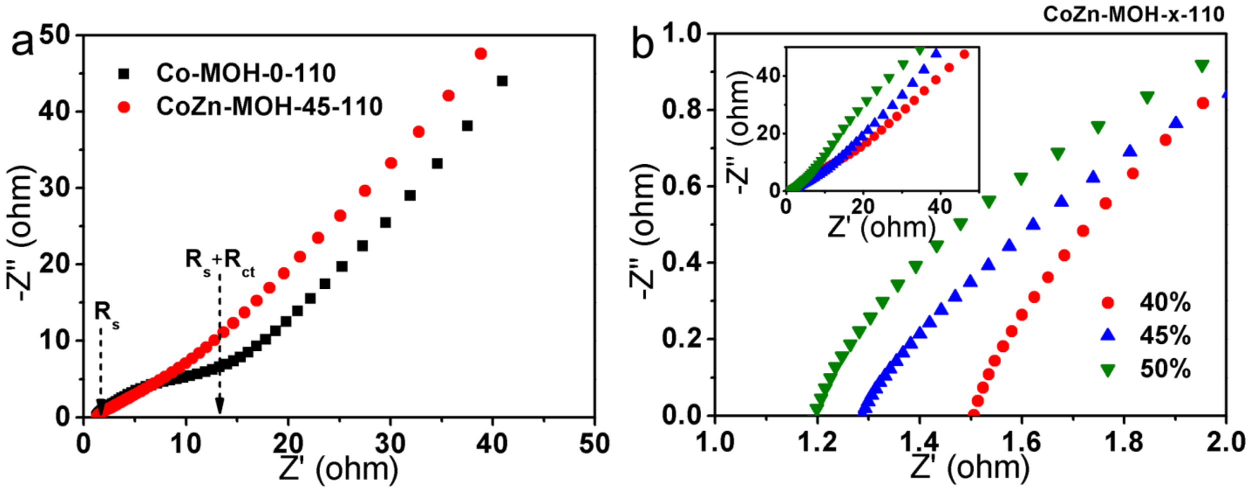

Figure S5. Comparisons of Nyquist plots: (**a**) Co-MOH-0-110 and CoZn-MOH-45-110 electrodes, (**b**) CoZn-MOH-x-110 electrodes prepared with different feeding molar percentage of zinc ion.

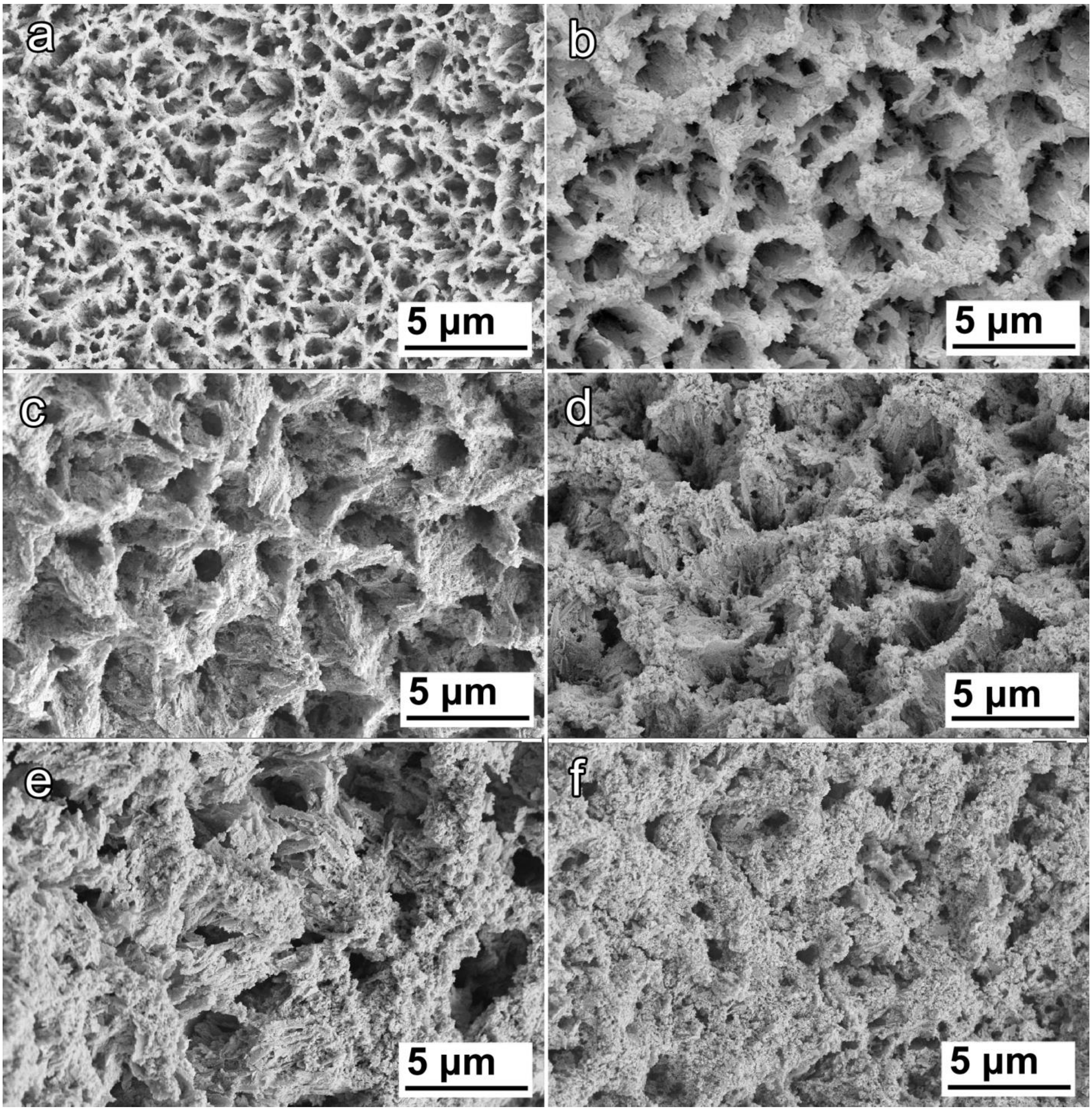

Figure S6. SEM images of CoZn-MOH-x-110 supported on nickel foam prepared with different feeding molar percentages of zinc ion: (**a**) 0%, (**b**) 33%, (**c**) 40%, (**d**) 45%, (**e**) 50%, and (**f**) 56%.

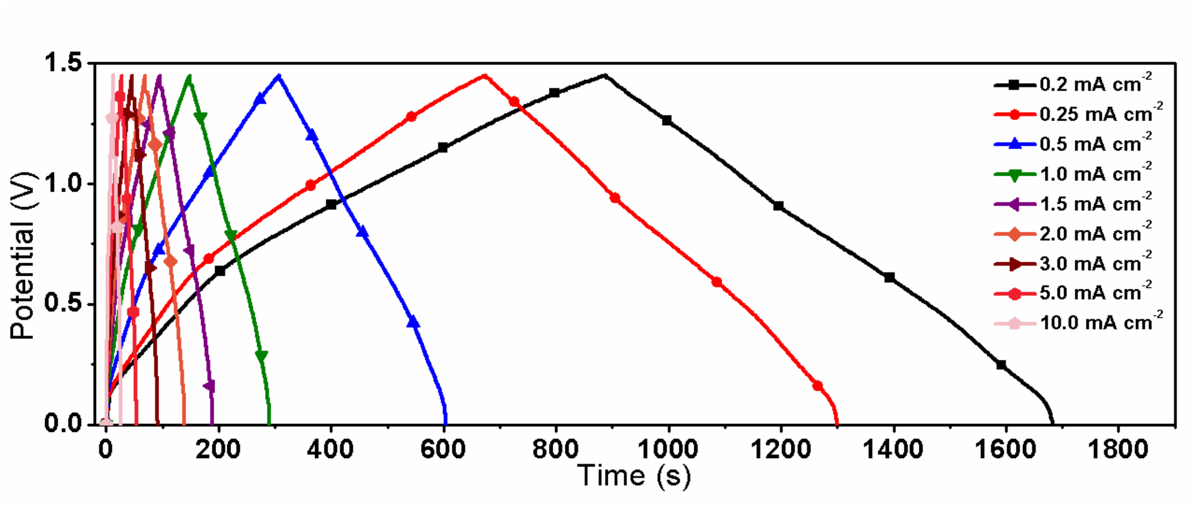

Figure S7. Galvanostatic CD curves of as-assembled CoZn-MOH-45-110//AC ASC.

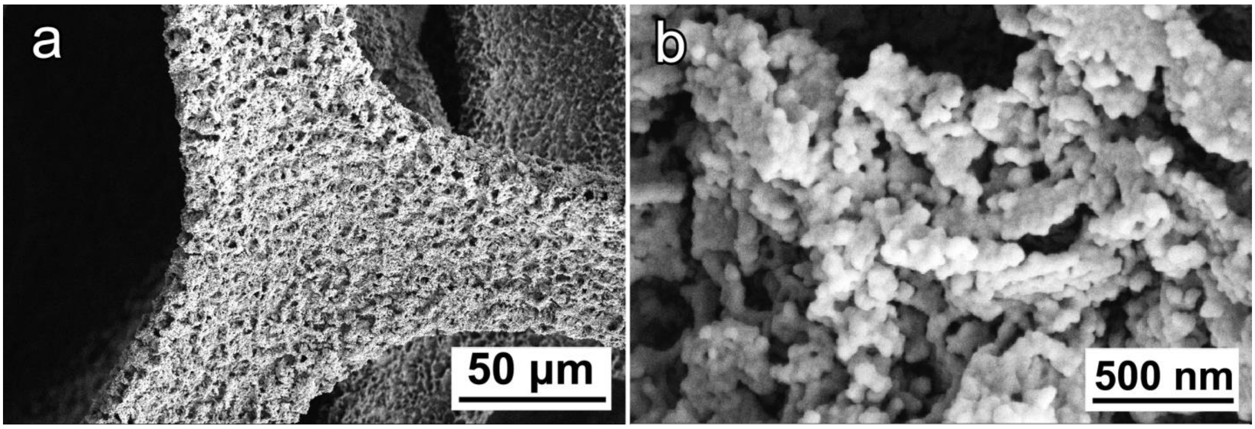

Figure S8. (**a**,**b**) SEM images of CoZn-MOH-45-110 electrode after the cycling performance test.
